# Root Canal Anatomy of Maxillary First Premolar by Microscopic Computed Tomography in a Chinese Adolescent Subpopulation

**DOI:** 10.1155/2019/4327046

**Published:** 2019-11-16

**Authors:** Xiaojing Liu, Meili Gao, Jianping Ruan, Qun Lu

**Affiliations:** ^1^Clinical Research Center of Shaanxi Province for Dental and Maxillofacial Diseases, Department of Preventive Dentistry, College of Stomatology, Xi'an Jiaotong University, XiWu Road 98, Xi'an 710004, Shaanxi Province, China; ^2^State Key Laboratory of Military Stomatology & National Clinical Research Center for Oral Diseases & Shaanxi Key Laboratory of Stomatology, Department of Operative Dentistry and Endodontics, School of Stomatology, Air Force Medical University, West Changle Road 145, Xi'an 710032, China; ^3^Department of Stomatology, Yulin First Hospital, Yuxi Avenue 93, Yulin 719000, Shaanxi Province, China; ^4^Key Laboratory of Shaanxi Province for Craniofacial Precision Medicine Research, Colleague of Stomatology, Xi'an Jiaotong University, Xi'an 710004, Shaanxi Province, China; ^5^Department of Biological Science and Engineering, The Key Laboratory of Biomedical Information Engineering of Ministry of Education, School of Life Science and Technology, Xi'an Jiaotong University, Xi'an 710049, China

## Abstract

**Objectives:**

To investigate the root morphology and root canal anatomy of maxillary first premolar using microscopic computed tomography (micro-CT).

**Methods:**

324 maxillary first premolars were collected and scanned. The root and canal diameter, canal wall thickness, root taper, and cross-sectional shapes were determined in the single root with 1 canal (SR1C), single root with 2 canals (SR2C), and 2 roots with 2 canals (2R2C) by micro-CT.

**Results:**

The results showed that single-rooted maxillary premolars were more common than other types. The incidence of SR1C, SR2C, and 2R2C reached 25%, 26.39%, and 26.39%, respectively. Root and canal diameters and canal wall thickness were decreased from coronal third to apical foramen. The three parameters and canal taper showed increases from buccal and palatal (BP) to mesiodistal (MD) aspects. The root canal tapers were smallest of the middle third level. The findings showed the different variations in 2R2C teeth. The root canal cross-sectional morphology in maxillary first premolars is complicated, including round, oval, long oval, flat canal, and irregular canal shapes. The distribution varied in different aspects.

**Conclusion:**

Root canal morphology showed a wide variation and complicated structure. The single-rooted teeth were more common in the Chinese adolescent population, and the majority of maxillary first premolars have two canals.

## 1. Introduction

Understanding and mastering of the appearance and internal structure of human teeth are essential for endodontists and researchers. Maxillary first premolars have unique anatomical features owing to its variation in root numbers and canal configuration. The anatomical structure of maxillary first premolars is complex, including bifurcated roots, great variations of root and canal morphology, and multiple canals [1–3]. These complex structures increase the difficulty of root canal treatment and postcore restoration, incompletion of root canal cleaning, and root canal lateral penetration or even root fracture [4]. Thus, a thorough understanding of the anatomical characteristics of the root canal system in the maxillary first premolar is essential for improving the success rate of root canal therapy and postcore restoration and reducing complications.

In recent years, microscopic computed tomography (micro-CT) has been used to study tooth morphology because of the ultrahigh resolution and high-precision three-dimensional images. Micro-CT is a noninvasive assessment that can vividly and intuitively reproduce three-dimensional images of teeth and root canals. It accurately measures tooth and is widely used to generate a series of cross-sectional images of a tooth [5–7]. Based on the micro-CT technology, it has already been reported that pulp cavities [8], three-dimensional images of root canal morphology [9], cross-sectional root canal shape (CSRCS) [10], buccal root with furcation groove [11], and root surface area [12] were observed and investigated in maxillary first premolars.

Teeth often indicate numerous external and internal changes with aging. The changes in root canal morphology over the course of a lifetime are a challenge to the clinician as they increase the difficulty of treatment. In adolescence, the root canal and its cavity are wide. The root canals become narrower with aging [13, 14]. Currently, the root canals by clearing technique and the percentage of each type of maxillary first premolar were reported [15]. The CBCT method was used to investigate condylar position and joint spaces [16] and buccal bone thickness [17] of maxillary first premolar in adolescent population. However, the detailed information of root canal anatomy of maxillary first premolar using micro-CT remained largely unknown in the Chinese adolescent population.

The aim of this work was to investigate the root canal anatomy of maxillary first premolars by using micro-CT in a Chinese adolescent subpopulation and to supply the further data of maxillary first premolar and to provide the reasonable suggestion for postpreparation in clinical treatment.

## 2. Materials and Methods

### 2.1. Samples

Samples used in this study were 324 maxillary first premolars collected from the adolescent population (15–25 years) which were removed due to orthodontics. The teeth were stored at the Orthodontic Department of Stomatology Hospital of Fourth Military Medical University in Xi'an, a northwestern province of China. Teeth selected in this study had met the following criteria: (1) the teeth are complete and have no fracture; (2) mature teeth with fully developed root; and (3) no root canal fillings, posts, or restoration. These teeth were cleaned in 3% hydrogen peroxide. After the removal of periodontal tissue and calculus, they were dried at room temperature. This research was approved by the Ethics Committee of Fourth Military Medical University. This study was conducted in accordance with the Helsinki Declaration.

### 2.2. Micro-CT Scanning and Analysis

All teeth were scanned using a micro-CT scanner (Siemens Inveon MM Gantry CT, Germany) with an isotropic resolution of 14.97 *μ*m and exposure time 500 ms at 80 kV and 500 *μ*A. The Mimics 10.01 (Materialise, Leuven, Belgium) was used for the 3-dimensional analysis.

**Figure 1:**
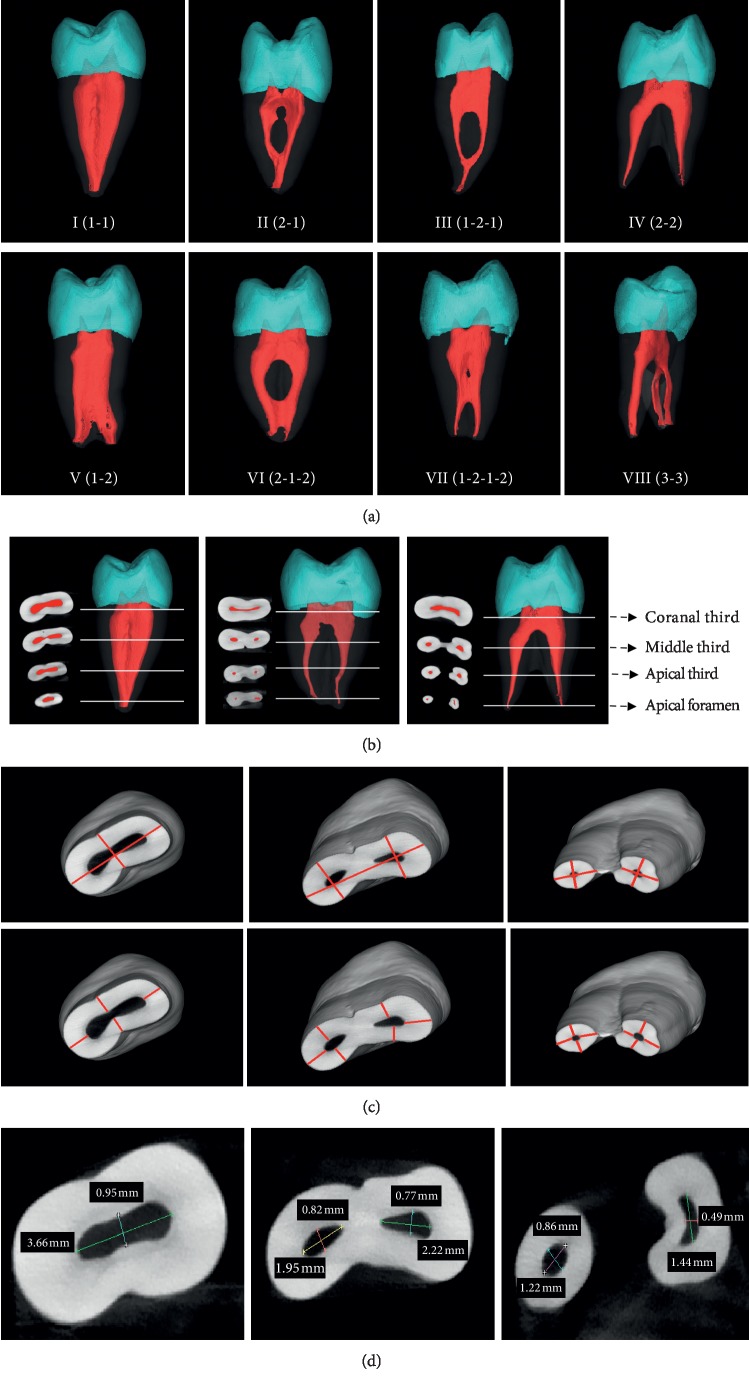
Three-dimensional reconstruction of a root canal by micro-CT of the examined maxillary first premolars (a), indication of coronal third, middle third, and apical third cross-sectional and apical foramen in cross section (b), measurement of root diameter (upper) and root canal wall thickness (lower) (c), and root canal diameter (d) of the further examined three maxillary first premolars, respectively.

Based on the 3D reconstruction images (Figure 1(a)), teeth with bifurcation or fusion of root canals were excluded. Following maxillary first premolars of single root with 1 canal (SR1C), single root with 2 canals (SR2C) and 2 roots with 2 canals (2R2C) were selected and analyzed additionally. Each type contained 70 teeth.

### 2.3. Root and Root Canal Analysis

In the cross-sectional images, the first slice where complete apical foramen appears was taken as the apex of the root canal and the first slice where enamel appears was taken as the neck of the root canal (i.e., the enamel-cementum junction). The distance from the root canal orifice to the apical foramen was set as the root canal length [18]. Then the root canals were divided into coronal third, middle third, and apical third (Figure 1(b)). The root diameter and root canal wall thickness (Figure 1(c)), root canal diameter (Figure 1(d)) of the three cross sections, and apical foramen in different directions were measured by using Mimics 10.01 software. All data measurements were performed by the two examiners. Each parameter was measured three times, and its average value was taken.

Based on the length of the root canal and diameter of crown and apex, the root canal taper (*C*) was calculated using the following formula: *C* = (*D* − *d*)/*L*, in which *D* and *d* represent the measured diameter of the crown and apex of the root canal segment and *L* is the length of the root canal length.

### 2.4. Assessment of Root Canal Shape

According to the classification criteria described by Jou et al., the shape of the root canal in four cross-sectional images was determined [19]. The quotient between the maximum and minimum width of root canals with the corresponding shape was as follows: a ratio 1 represents a round, up to 2 an oval, between 2 and 4 a long oval, more than 4 a flatted root canal, and more than 5 an irregular shape.

### 2.5. Statistical Analysis

The data were analyzed using SPSS 17.0 statistical software to carry out one-way ANOVA. The SNK-*q* test was used to compare the two groups, and a value of *P* < 0.05 was statistically significant.

## 3. Results

### 3.1. Root Canal Morphology

In the examined 324 maxillary first premolars, according to Vertucci's classification, the root canal morphology was established using micro-CT and shown in Figure 1(a).  Table 1 shows that the frequency of the single-rooted teeth was highest (72.22%), and two-rooted teeth followed (26.54%), and triple-rooted was only 1.23%. Additionally, the incidence of single root with 1 canal (SR1C), single root with 2 canals (SR2C), and 2 roots with 2 canals (2R2C) was 81 (25%), 85 (26.23%), and 86 (26.54%), respectively. In the following investigation, we measured the three maxillary first premolars.

### 3.2. Root Diameters

Root diameters of the three maxillary first premolars were measured firstly and are shown in Table 2. Root diameters were decreased from cervical third to apical foramen except those in 1/3 BP at the middle third level of single root with 1 canal and 2 roots with 2 canals. Similarly, the root diameters indicated increases from the buccal and palatal diameter (BP) to mesiodistal diameter (MD) except those in apical third and apical foramen of buccal roots of single root with 2 canals.

### 3.3. Root Canal Wall Thickness

The root canal wall thickness at different levels is shown in Table 3. Generally, the wall thickness was decreased from the CEJ to the apex. The buccal and palatal walls of the single rooted with 1 canal maxillary first premolar were significantly thicker (*P* < 0.05) than the distal and mesial walls at each portion. No significant difference in wall thickness was observed at different aspects in these single root with 2 canals teeth. The wall thickness of the palatal wall of the buccal root was markedly less than the mesio- and distal-aspects among these 2 roots with 2 canals teeth (*P* < 0.05). The average wall thickness of the palatal aspect of the apical one-third of the buccal root is just 0.5 mm. This result corresponds to the result reported by Paola; he found that the average wall thickness is on average less than 1 mm (discussion). The palatal wall of the palatal root was significantly thicker than the distal and mesial walls at the level of the cervical third and middle third, and the difference was statistically significant (*P* < 0.05).

### 3.4. Root Canal Diameters

As shown in Table 4, the root canal diameters of maxillary first premolars were increasing from the apical foramen to cervical third. The root canal diameter of single root with 1 canal, single root with 2 canals, and 2 roots with 2 canals in buccal-palatal direction was significantly greater than that in mesial-distal direction (*P* < 0.05) except that in the apical foramen. However, the significant smaller diameter was observed (*P* < 0.05) at apical third in the buccal-palatal direction of the buccal canal of 2 roots with 2 canals teeth.

### 3.5. Root Canal Taper

The root canal tapers of the examined teeth (Table 5) were the smallest of the middle third. Additionally, the root canal tapers were significantly larger (*P* < 0.05) at the buccal and palatal aspects than those in the corresponding mesiodistal aspect except those at the apical third of the buccal canal of single root with 2 canals and palatal canal of 2 roots with 2 canals. On the contrary, the root taper at the apical third of the buccal and palatal aspect was significantly smaller (*P* < 0.05) than that in the mesiodistal aspect.

### 3.6. Root Canal Cross-Sectional Shape

**Figure 2:**
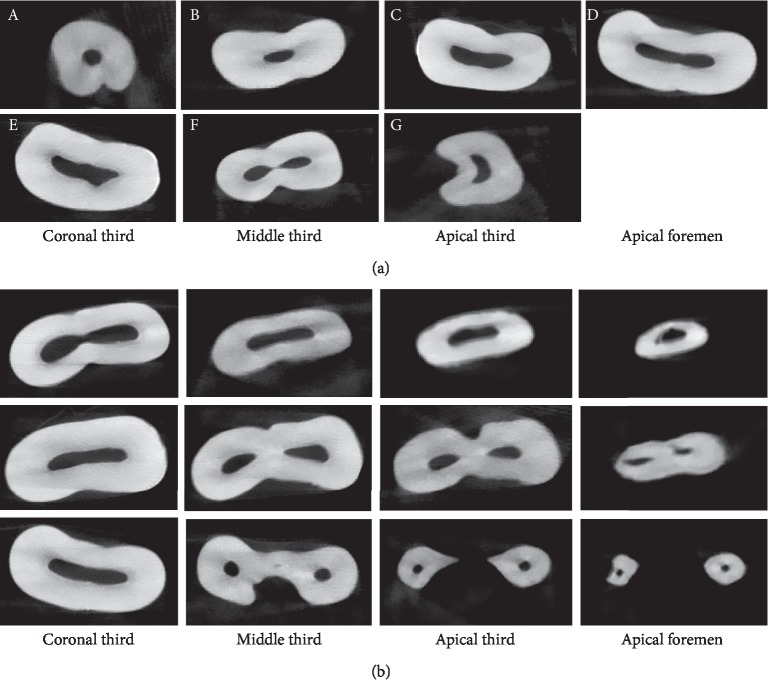
Cross-sectional root canal shape (a) and images (b) of maxillary first premolar. A, round; B, oval; C, long oval; D, flat; E and F, irregular shape; E, triangle; F, “8” figure; G, semilunar.

The root canal cross-sectional morphology in maxillary first premolars is complicated, including round canal, oval canal, long oval canal, flat canal, and irregular canal (Figure 2, Table 6). Triangle, “8” figure and semilunar shapes were found in irregular canals (Figure 2). At the cervical third level, long oval, flat, and irregular shapes (90%–100%) were found in the three types of maxillary first premolars. At the middle third level, oval and long oval shapes (70%–90%) were observed in single-rooted maxillary first premolars. In 2-rooted maxillary first premolars, oval and irregular shapes (90%) were found at the middle third of the buccal canal, and long, flat, and irregular shapes (100%) were observed at the middle third of the palatal canal. Round and oval shapes (60–80%) were found at apical third and apical foramen levels of in single-rooted maxillary first premolars. Additionally, for the buccal canal, oval and irregular shapes (80%) at apical third level and round and irregular shapes (90%) were observed in 2 roots with 2 canals maxillary first premolars. For palatal canal, besides irregular shape in both levels, oval and flat shapes at apical third level and round and oval shapes at apical foramen were observed in the examined 2-rooted maxillary first premolars.

## 4. Discussion

Root canal morphology of the maxillary first premolar showed a wide variation, and the complexity of the root canal system brings great challenges to root canal treatment [1–4]. It is very important for the clinician to be familiarized with the canal morphology. For root canal anatomy aberrations assay, previous studies have reported that for canal staining and tooth clearing, periapical radiographic radiographs were used. However, these techniques are invasive or allow only 2-dimensional (2D) analysis [11]. Cone-beam computed tomography (CBCT) is noninvasive and can provide improved accuracy and higher resolution [21], while the advantage of micro-CT can show internal and external dental anatomies [9]. Thus, in this study, to further provide detailed information of the internal anatomical data in root canal, we established eight types of root canal morphologies using micro-CT and evaluated the root diameter, root canal diameter, canal wall thickness, canal diameter, and root canal shape of extracted teeth from Chinese adolescent subpopulation.

Awawdeh and his colleagues have reported the frequency of one root (30.8%) and two roots (68.4%) in Jordanian maxillary first premolars [22]. In an Egyptian subpopulation study, more than half had two roots, and about 45% had a single root in maxillary first premolars [23]. Our study presented a higher prevalence of single-rooted teeth and a lower prevalence of two-rooted teeth than these reported studies. Although the results about the root number vary a lot, we can speculate that single-rooted maxillary premolars were more common in the Chinese adolescent population, for the prevalence exceeded more than half. The frequency of one root was 57.36% in 422 maxillary first premolars collected from the adolescent population ranging from 12–26 years [15], which is relative lower than 72.22% of our data. In a total of 300 CBCT images involving maxillary first premolar teeth from 241 patients study, the frequency of one root was 66% [24]. Of all the maxillary first premolars, teeth with two canals were the most common (171/324). The prevalence of teeth with three canals was 1.23% (4/324) which is similar to the prevalence (1.4%) of Egyptian subpopulation study of maxillary premolar teeth [23]. Though the prevalence of teeth with three canals was very small, it should not be forgotten to miss any canals. Simultaneously, the incidence of single root with 1 canal (SR1C), single root with 2 canals (SR2C), and 2 roots with 2 canals (2R2C) indicated higher incidence than other types maxillary first premolars. The canal anatomy of three types of maxillary first premolars was further analyzed.

Root canal diameter was called as “the forgotten dimension” which was underestimated [19]. Grande et al. reported that the buccolingual (BL) diameter was greater than MD in both root and canal in a 30 single-rooted premolars [25]. The largest canal diameter of the maxillary first premolar exhibited as 1.26 ± 0.26 mm in a previous reported study [26]. In our study, the largest root canal diameter was 1.15 ± 0.30 mm in the maxillary first premolar. Our findings were consistent with these reports. However, the root and canal diameters of the buccal side at BL levels were smaller than that at MD levels in maxillary first premolar with two canals in our study. The more likely it is that the furcation groove exists on the palatal aspect of the buccal root. It has been reported that the buccal-palatal canal diameter of the buccal root is negatively correlated with the furcation groove on the palatal aspect of the buccal root [27].

The bifurcated maxillary first premolar has the unique anatomic landmark. In our study, the wall thickness was larger in the buccal and palatal sides in single-rooted maxillary first premolar with one canal. This is in agreement with Grande et al. [25]. However, in maxillary first premolar with 2 canals, no statistical or opposite smaller canal thickness was indicated in our investigation. The root and canal diameter in the present study imply the inconsistency of the wall thickness. The wall thickness was affected by the prevalence of the furcation groove in maxillary first premolars [11, 27]. The average wall thickness of the palatal aspect of the apical one-third of the buccal root is just 0.5 mm on the account of the existence of the furcation groove. In a study of forty-two bifurcated maxillary first premolars of the Chinese population, Li et al. reported that the minimum wall thickness of the apical third of the buccal root was just 0.26 mm [11]. This difference may be due to the different Chinese populations examined. Thus, this portion is a danger zone where perforation may easily occur and the palatal aspect of the buccal should not be removed excessively. Meanwhile, this portion may be a predilection site of root fracture. A residual dentin thickness of less than 1 mm jeopardizes root integrity. Furthermore, the inconsistency of the wall thickness leads to the fact that tensile stress was concentrated. So, this portion was susceptible to vertical root fracture [28, 29].

There are few studies to investigate the cross-sectional root canal shape by micro-CT. Rechenberg and Paque´ have found that the cross-sectional root canal shape was almost round in two-rooted maxillary first premolars with one straight canal per root maxillary premolars [10]. We here found the round shape is mainly distributed in apical foremen. This may be due to the different types examined. We additionally provided the evidence that oval and long-oval shaped mainly distributed at the middle third and apical third. Simultaneously, long, flat, and irregular shapes were mainly located at coronal third. These results provide the evidence of the distribution dependence on different cross-sectional levels of maxillary first premolars. The cross-sectional root canal shape is closely related to root canal preparation and the effect of filling material. The determination of width in root canal preparation was more complicated because of the variation of cross-sectional root canal shape [30, 31]. Root canal diameter and taper play a crucial role in the selection of preparation instruments [31]. It should select the corresponding root canal preparation instrument according to the taper values of different parts of the root canal in maxillary first premolars. The established information of root taper in our study may provide the preparation instrument in clinical treatment.

After 15 years of age, the predictability of orthopedic expansion is greatly decreased in adolescents [32]. To the best of our knowledge, no study has so far evaluated root canal diameter and wall thickness of maxillary premolars of in a Chinese adolescent subpopulation using micro-CT. Root canal anatomy is indeed susceptible to changes over the years. Root canal system morphology may become completely calcifed with secondary dentine with aging [13, 14]. For example, diameter root canal orifice of maxillary first premolars is larger in 20s than that in 40s [8], and the widest root canals of maxillary central teeth were in the 15–24 year age group [13]. The data of maxillary first premolars presented here may provide the appearance and internal structure of human maxillary first premolars in adolescents.

This study has provided a detailed description of the root and canal morphologies of maxillary first premolar by micro-CT in a Chinese adolescent subpopulation. However, this study has some limitations. First, the limitation of our study was relative small sample size used in micro-CT analysis, especially considering the symmetry of root canal anatomy [33], so the research should be deepened by expanding the sample size to the entire Chinese population, including adolescent population in different areas of China in the future. Second, the studied teeth sample was mainly collected from young patients (15–25 y). Actually the size of the inner root canal will decrease with aging. The data and information derived in this study may be inapplicable for other age people. The more information of root canal diameter, thickness of root canal walls, apical cementum apposition, and even the taper in other age people should be further studied to improve and perfect the root canal morphological characteristics.

## 5. Conclusion

In short, root canal morphology of the maxillary first premolar showed a wide variation, including root diameter, root canal diameter, canal wall thickness, canal diameter, and cross-sectional root canal shape. The single-rooted teeth were more common in the Chinese adolescent subpopulation, and the majority of maxillary first premolars have two canals. Of the 2 roots with 2 canals maxillary first premolars, the furcation groove on the palatal aspect of the buccal root has a great influence on the treatment and prognosis. Based on these anatomical structures, our paper also provides reference for the clinical treatment for maxillary first premolar-associated diseases in the future.

## Figures and Tables

**Table 1:** Frequency of root canal morphology of maxillary first premolars according to Vertucci's [20] classification.

Roots	No. (%)	Root canal types							
		I	II	III	IV	V	VI	VII	VIII
		(1-1)	(2-1)	(1-2-1)	(2-2)	(1-2)	(2-1-2)	(1-2-1-2)	(3-3)
One root	234 (72.22%)	81	27	9	85	18	9	5	0
Two roots	86 (26.54%)	0	0	0	86	0	0	0	0
Three roots	4 (1.23%)	0	0	0	0	0	0	0	4
Total	324	81 (25%)	27 (8.33%)	9 (2.78%)	171 (52.78%)	18 (5.56%)	9 (2.78%)	5 (1.54%)	4 (1.23%)

**Table 2:** Root diameters in different levels of the three maxillary first premolars (mean ± SD, mm, *n* = 70).

Groups	Cervical third	Middle third	Apical third	Apical foramen
SR1C				
BP	8.02 ± 2.73	6.78 ± 3.04	4.98 ± 2.89	2.23 ± 0.45
1/3 BP	2.67 ± 0.91	2.26 ± 1.01	1.66 ± 0.96	0.74 ± 0.15
MD	4.14 ± 0.13	2.61 ± 1.11	2.13 ± 0.73	1.46 ± 0.67
1/3 MD	1.47 ± 0.04	0.87 ± 0.37	0.71 ± 0.24	0.49 ± 0.22

SR2C				
BP	8.91 ± 2.68	8.20 ± 2.71	7.30 ± 1.55	2.10 ± 0.55
1/3 BP	2.97 ± 0.89	6.73 ± 0.90	2.43 ± 0.31	0.70 ± 0.18
MD (buccal)	5.83 ± 1.89	4.17 ± 1.93	3.28 ± 0.87	1.86 ± 0.13
1/3 MD (buccal)	1.94 ± 0.63	1.39 ± 0.64	1.09 ± 0.29	0.62 ± 0.04
MD (palatal)	5.67 ± 2.33	4.01 ± 0.83	2.75 ± 0.35	1.85 ± 0.33
1/3 MD (palatal)	1.89 ± 0.78	1.34 ± 0.27	0.90 ± 0.11	0.61 ± 0.11

2R2C				
Buccal roots				
BP	8.84 ± 3.00	8.24 ± 3.12	2.61 ± 0.54	1.30 ± 0.34
1/3 BP	2.95 ± 1.00	3.74 ± 1.04	0.87 ± 0.18	0.44 ± 0.11
MD	4.62 ± 1.01	3.74 ± 1.13	2.90 ± 0.59	1.33 ± 0.31
1/3 MD	1.54 ± 0.34	1.24 ± 0.37	0.97 ± 0.19	0.44 ± 0.10

Palatal roots				
BP	8.84 ± 3.35	8.24 ± 3.41	2.93 ± 0.71	1.80 ± 0.21
1/3 BP	2.95 ± 1.12	3.74 ± 1.13	0.98 ± 0.23	0.60 ± 0.07
MD	4.44 ± 1.11	3.22 ± 0.57	2.68 ± 0.48	1.46 ± 0.32
1/3 MD	1.48 ± 0.37	1.41 ± 0.19	0.90 ± 0.16	0.49 ± 0.11

SR1C, single root with 1 canal; SR2C, single root with 2 canals; 2R2C, 2 roots with 2 canals; BP, buccal and palatal diameters; MD, mesiodistal diameter.

**Table 3:** Root canal wall thickness in different levels of maxillary first premolar (mean ± SD, mm, *n* = 70).

Groups	Cervical third	Middle third	Apical third	Apical foramen
SR1C				
B	2.32 ± 0.12^*∗*^^,#^	2.19 ± 0.23^*∗*^^,#^	1.76 ± 0.46^*∗*^^,#^	0.89 ± 0.25
P	2.43 ± 0.44^*∗*^^,#^	2.36 ± 0.38^*∗*^^,#^	2.01 ± 0.13^*∗*^^,#^	0.73 ± 0.19
M	1.52 ± 0.35	1.00 ± 0.12	0.87 ± 0.32	0.66 ± 0.15
D	1.84 ± 0.45	1.12 ± 0.24	0.90 ± 0.35	0.52 ± 0.16

SR2C				
Buccal canal				
B	2.33 ± 0.32	1.91 ± 0.34	1.37 ± 0.50	0.58 ± 0.12
M	2.08 ± 0.12	1.55 ± 0.16	1.33 ± 0.67	0.57 ± 0.10
D	1.99 ± 0.33	1.68 ± 0.61	1.34 ± 0.43	0.65 ± 0.24

Palatal canal				
P	2.53 ± 0.30	1.86 ± 0.36	1.33 ± 0.62	0.37 ± 0.08
M	1.83 ± 0.38	1.67 ± 0.61	1.27 ± 0.33	0.63 ± 0.11
D	1.90 ± 0.17	1.77 ± 0.23	1.17 ± 0.26	0.32 ± 0.09

2R2C				
Buccal canal				
B	2.21 ± 0.34	1.60 ± 0.60	1.08 ± 0.29	0.47 ± 0.09
P	—	—	0.5 ± 0.13^*∗*^^,#^	0.3 ± 0.07^*∗*^^,#^
M	1.75 ± 0.40	1.32 ± 0.53	1.07 ± 0.35	0.49 ± 0.10
D	1.75 ± 0.53	1.46 ± 0.33	1.07 ± 0.33	0.49 ± 0.12

Palatal canal				
B	—	—	1.09 ± 0.31	0.64 ± 0.12
P	2.52 ± 0.33^*∗*^^,#^	1.83 ± 0.31^*∗*^^,#^	1.10 ± 0.30	0.52 ± 0.15
M	1.49 ± 0.19	1.07 ± 0.35	1.07 ± 0.22	0.52 ± 0.21
D	1.80 ± 0.54	1.15 ± 0.28	1.07 ± 0.18	0.49 ± 0.08

^*∗*^represents vs. corresponding M, *P* < 0.05;# represents vs. corresponding D, *P* < 0.05. SR1C, single root with 1 canal; SR2C, single root with 2 canals; 2R2C, 2 roots with 2 canals; B, buccal aspect; P, palatal aspect; M, mesio-aspect; D, distal-aspect.

**Table 4:** Root canal diameter in different levels of maxillary first premolar (mean ± SD, mm, *n* = 70).

Groups	Cervical third	Middle third	Apical third	Apical foramen
SR1C				
BP	3.34 ± 0.85^a^	2.16 ± 0.61^a^	1.57 ± 0.32^a^	0.59 ± 0.18^a^
MD	0.75 ± 0.23	0.42 ± 0.13	0.42 ± 0.10	0.3 ± 0.11

SR2C				
Buccal canal				
BP	—	1.38 ± 0.21^a^	0.87 ± 0.25^a^	0.62 ± 0.17^a^
MD	0.98 ± 0.30	1.01 ± 0.31	0.59 ± 0.09	0.44 ± 0.09

Palatal canal				
BP	—	1.09 ± 0.21^a^	0.57 ± 0.14^a^	0.21 ± 0.05
MD	0.82 ± 0.31	0.66 ± 0.11	0.35 ± 0.06	0.21 ± 0.02

2R2C				
Buccal canal				
BP	—	1.66 ± 0.23^a^	0.38 ± 0.14^a^	0.12 ± 0.06^a^
MD	1.12 ± 0.30	0.96 ± 0.28	0.77 ± 0.13	0.50 ± 0.09

Palatal canal				
BP	—	1.25 ± 0.21^a^	0.75 ± 0.11^a^	0.65 ± 0.10^a^
MD	1.15 ± 0.30	0.93 ± 0.23	0.55 ± 0.09	0.33 ± 0.06

a represents vs. the corresponding MD, *P* < 0.05. SR1C, single root with 1 canal; SR2C, single root with 2 canals; 2R2C, 2 roots with 2 canals; BP, buccal and palatal diameters; MD, mesiodistal diameter.

**Table 5:** Root canal taper in different levels of maxillary first premolar (mean ± SD, mm, *n* = 70).

Groups	Coronal third	Middle third	Apical third
SR1C			
BP	0.30 ± 0.082^a^	0.16 ± 0.042^a^	0.25 ± 0.126^a^
MD	0.12 ± 0.033	0.07 ± 0.035	0.12 ± 0.041

SR2C			
Buccal canal			
BP	—	0.20 ± 0.091^a^	0.09 ± 0.027
MD	0.15 ± 0.026	0.14 ± 0.081	0.09 ± 0.039

Palatal canal			
BP	—	0.16 ± 0.044^a^	0.10 ± 0.051^a^
MD	0.12 ± 0.039	0.05 ± 0.027	0.03 ± 0.018

2R2C			
Buccal canal			
BP	—	0.17 ± 0.043^a^	0.11 ± 0.022^a^
MD	0.14 ± 0.041	0.07 ± 0.027	0.06 ± 0.018

Palatal canal			
BP	—	0.15 ± 0.031^a^	0.03 ± 0.016^a^
MD	0.12 ± 0.032	0.10 ± 0.042	0.06 ± 0.013

a represents vs. the corresponding MD, *P* < 0.05. SR1C, single root with 1 canal; SR2C, single root with 2 canals; 2R2C, 2 roots with 2 canals; BP, buccal and palatal aspects; MD, mesiodistal aspect.

**Table 6:** Distribution of root canal shape in the cross-sectional image of maxillary first premolars.

Group	Round	Oval	Long oval	Flat	Irregular shape	Total
SR1C						
Coronal third	0	0	21 (30%)	35 (50%)	14 (20%)	70
Middle third	0	28 (40%)	21 (30%)	7 (10%)	14 (20%)	70
Apical third	14 (20%)	28 (40%)	21 (30%)	0	7 (10%)	70
Apical foremen	35 (50%)	21 (30%)	7 (10%)	0	7 (10%)	70

SR2C						
Buccal canal						
Coronal third	0	7 (10%)	21 (30%)	28 (40%)	14 (20%)	70
Middle third	7 (10%)	35 (50%)	21 (30%)	0	7 (10%)	70
Apical third	21 (30%)	28 (40%)	14 (20%)	0	7 (10%)	70
Apical foremen	35 (50%)	21 (30%)	0	0	14 (20%)	70

Palatal canal						
Coronal third	0	7 (10%)	21 (30%)	28 (40%)	14 (20%)	70
Middle third	0	35 (50%)	28 (40%)	7 (10%)	0	70
Apical third	7 (10%)	21 (30%)	14 (20%)	7 (10%)	21 (30%)	70
Apical foremen	35 (50%)	28 (40%)	0	0	7 (10%)	70

2R2C						
Buccal canal						
Coronal third	0	0	7 (10%)	42 (60%)	21 (30%)	70
Middle third	0	28 (40%)	7 (10%)	0	35 (50%)	70
Apical third	7 (10%)	21 (30%)	7 (10%)	0	35 (50%)	70
Apical foremen	35 (50%)	7 (10%)	0	0	28 (40%)	70

Palatal canal						
Coronal third	0	0	7 (10%)	42 (60%)	21 (30%)	70
Middle third	0	0	28 (40%)	28 (40%)	14 (20%)	70
Apical third	0	35 (50%)	21 (30%)	7 (10%)	14 (20%)	70
Apical foremen	28 (40%)	21 (30%)	0	0	21 (30%)	70

## Data Availability

The micro-CT images data used to support the findings of this study are restricted by the local institutional review board at the Air Force Medical University in order to protect patients' privacy.
